# Slope stability analysis considering the strength anisotropy of *c*-*φ* soil

**DOI:** 10.1038/s41598-022-20819-y

**Published:** 2022-11-01

**Authors:** Yi He, Zhi Li, Wenfa Wang, Ran Yuan, Xiaoyan Zhao, Nikolaos Nikitas

**Affiliations:** 1 Key Laboratory of High-Speed Railway Engineering, Ministry of Education, Southwest Jiaotong University, Chengdu, 610031 China; 2 Faculty of Geosciences and Environmental Engineering, Southwest Jiaotong University, Chengdu, 611756 China; 3 Key Laboratory of Transportation Tunnel Engineering, Southwest Jiaotong University, Chengdu, 610031 China; 4 School of Civil Engineering, University of Leeds, Leeds, LS2 9JT UK

**Keywords:** Natural hazards, Civil engineering

## Abstract

In traditional slope stability analyses, soil is usually approximated as isotropic. However, naturally cohesive soil deposits are inherently anisotropic, primarily due to the directional arrangement of soil particles during their deposition process. In this paper, a generalized anisotropic constitutive model for *c*–*φ* soil is introduced to evaluate the influence of varying shear strength on slope stability. In this model, the initial strength anisotropy is defined by the variety of friction angles to the direction of the principle stress. This model is utilized by two approaches to estimate the slope stability. Firstly, the upper bound limit analysis solution for slope stability is developed, and the safety factor of the slopes is studied. Secondly, this model is coupled with the finite element method to get insight of the influence of anisotropy on slope stability. One typical slope case of slope is studied by numerical analyses. It is found that the slope stability is largely overestimated when the strength anisotropy is ignored, and the overestimation, in terms of safety factors, can reach up to 32.9%. The complex interrelations between the degree of anisotropy and evolution of the ensuing safety factor are revealed by a series of parametric studies in terms of different degrees of anisotropy.

## Introduction

Slope failure has been a major cause of human and material losses, and for this reason it has become a popular subject for considerable, concerted research^1–8^. Although various modelling details play a significant role in slope stability analyses, one of the key aspects is the constitutive modelling employed to describe the mechanical behavior of the soil^9^. A wide gap still exists between research and practice, because typical simple constitutive models used in every-day engineering are not fully capable of describing the complex mechanical behavior of real geomaterials. As such, deformation and safety factor (*F*_s_) predictions may not meet the accuracy requirements. Although the anisotropic mechanical behavior of natural soils has been discussed widely, in traditional analyses, following an oversimplification rationale, soils are most often regarded as isotropic. To recover prediction accuracy for a number of problems in soil mechanics, slope stability included, the shear anisotropy of soil needs to be explicitly considered for a more faithful replication of the soil medium^10–13^.

The strength anisotropies of soils are usually classified into two types, the inherent (initial) and induced ones. The former relates to the influence of soil self-weight and tectonic stresses in the process of natural deposition, which results in directionally varying shear strength^13,14^. The latter stems from the rearrangement of particles and the associated change in the void space distribution under external loads^15^. Generally, in the consolidation direction (usually the vertical direction), soils exhibit higher shear strength than those in the other directions. This has been demonstrated by the finding that the triaxial compression strength is typically higher than the triaxial extensive strength for the same specimens^16,17^.

Strength anisotropy of soils has considerable bearing on the limited dependability of many stability analyses, and the action of slopes accounting for anisotropy is a subject that incentivizes relevant explicit studies. In the last decades, many studies concentrated on the strength anisotropy of pure clay (*φ* = 0), because the phenomenon that cohesion varies with direction has been demonstrated by the vane shear test, and it is possible to measure the values of the varied cohesions of the remolded soil specimens through laboratory test^18,19^. For instance, in total 139 unconfined compression tests were carried out by Lo^20^ to study the variation of cohesion with respect to direction, and the statistical results were applied to the slope stability analysis associating with the limit equilibrium method. The results showed that the influence of anisotropy on the stability is significant for flat slopes but smaller for steep slopes. Based on the Casagrande and Carillo’s concept^21^, the effects of cohesion anisotropy on slope stability for *c*-*φ* soil has been studied by Chen^22^ using the limit analysis method. It was found that effects of strength anisotropy on the stability number for *c*-*φ* soil increases with the decrease in the slope angle, and this effect gets more pronounced while the friction angle of the soil is greater than 10°. Al-Karni and Al-Shamrani^23^ introduced the cohesion anisotropy concept into the Bishop simplified method of slices. They indicated that the geometry of the slip surface is only slightly affected by the degree of anisotropy in the soil cohesion. The accuracy of the safety factor of the slopes depended on a large number of laboratory test results when the shear anisotropy is considered. In order to get more accuracy with less test datum for slope stability analyses, Su and Liao^24^ proposed a strength criterion to analyze the shear strength of undrained clay along the failure surface, where the anisotropic slope stability problem can be reduced to isotropic counterparts as a particular case.

**Figure 1:**
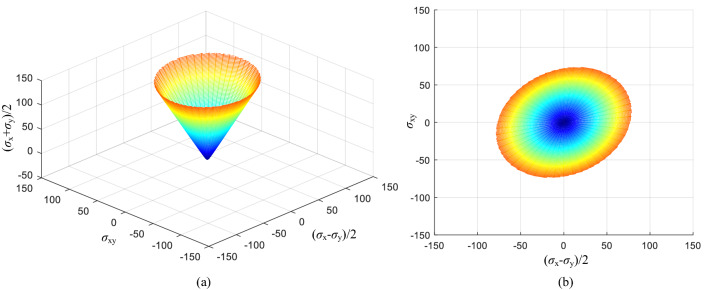
General yield surface: (**a**) three dimensional stress space [(*σ*_x_ + *σ*_y_)/2, (*σ*_x_−*σ*_y_)/2, *σ*_xy_], and (**b**) the cross-section in the stress space of [(*σ*_x_−*σ*_y_)/2, *σ*_xy_].

The above researches focused on the cohesion anisotropy and its corresponding effects on the slope stability, it is simply because the directionally varying cohesion can be easily measured through in-situ test or laboratory test. However, the effects of the internal friction angle varying with the loading direction for slope stability problems, have not been adequately studied^19,25^, despite the fact that such effects have been widely discussed by many researches involving porous media^14,26^. Some attempts have been made to study this specific issue^13,15,27–31^; nevertheless, limited by the complex theory, the relevant soil strength parameters and anisotropic parameters are not easy to be measured, thus it is not convenient to be applied into the practical engineering. Until recent years, the application of hollow cylinder tests makes it possible to measure the variation of friction angle more accurately, and also enables validation of theoretical anisotropic models (Fig. 1)^14,32^. For natural deposited slopes, the directionally arranged soil particles may cause the initial strength anisotropy of granular materials. However, to what extent the varying internal friction angles will impact the slope stability has not been fully studied. In addition, for convenience in practice, a simply constitutive model where the strength parameters and the anisotropy index can be easily measured is still needed.

In this paper, a newly proposed general plane-strain, elastic perfectly plastic constitutive model incorporating initial strength anisotropy is reviewed. Hooke's law pertains in the elastic regime. The yield criterion, extended from the classical isotropic Mohr–Coulomb yield criterion, takes the initial strength anisotropy into account. The initial strength anisotropy is considered by the variation of the internal friction angle with the direction of the principal stresses and an associated flow rule is utilized. The strength anisotropy is defined by two parameters, i.e., *n* and *β*, which can be easily measured through a hollow cylinder test. The developed constitutive model is firstly introduced into the limit analysis (LA) method. The stability factor (*γH*/*c*) of slopes is analyzed based on the kinematic theory within the framework of limit analysis. The effects of anisotropic parameters on the stability factor are evaluated. Further, the slope safety factor (*F*_s_) is studied as well. For the purpose of comparison, the anisotropic yield criterion is applied in conjunction with the finite element method (FEM) where a shear strength reduction approach (SSRA) is used to bring the slopes to the point of failure (limit state). The explicit modified Euler algorithm with stress corrections^33,34^ is applied to integrate the elastic–plastic stress strain relationship towards calculating the problem stresses. The failure surface and the *F*_s_ obtained by the two methods are compared with each other. The influence of strength anisotropy on critical slip surfaces and *F*_s_ of slopes are scrutinized, and the explicit sensitivity of the modelled anisotropic parameters are reviewed by FEM.

## The anisotropic yield criterion

The anisotropic yield criterion employed was first proposed by Yuan et al^25^ to describe the friction angle variation with principal stress orientation. The shape of a resulting yield surface is shown in Fig. 1. Conventionally, throughout this paper, negative stress denotes compression. For coherence, a brief introduction of the anisotropic criterion derivation follows.

**Figure 2:**
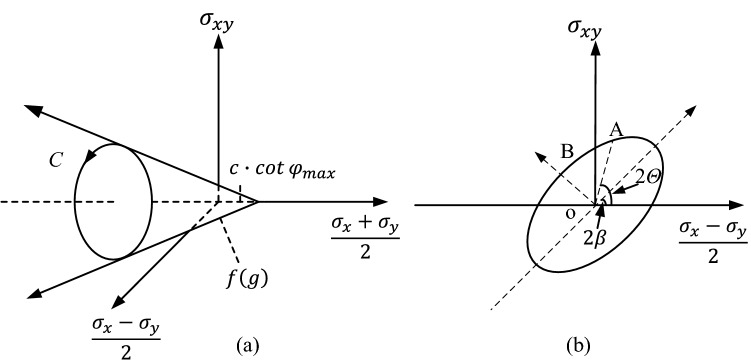
Yield surface in the stress space of (**a**) three dimension; (**b**) (*σ*_x_−*σ*_y_)/2, *σ*_xy_.

Booker and Davis^35^ proposed the hypothesis that the anisotropic yield surface of granular materials can be characterized by the hydrostatic pressure *p* and the principal stress direction *Θ*. Yuan et al.^25^ as per Booker and Davis^35^, considered the yield surface in the [(*σ*_x_−*σ*_y_)/2, *σ*_*x*y_] space as an ellipse (Fig. 2) demonstrating that the anisotropic yield criterion can be explicitly given by

1 $$ f(\sigma_{x} ,\sigma_{y} ,\sigma_{xy} ) = R + F(p,\Theta ) = 0, $$

with

1a $$ R = \frac{1}{2}\sqrt {(\sigma_{x} - \sigma_{y} )^{2} + 4\sigma_{xy}^{2} } , $$

1b $$ F(p,\Theta ) = (p - c\cot \phi_{\max } ) \cdot \sin \phi (\Theta ), $$

1c $$ p = \frac{1}{2}(\sigma_{x} + \sigma_{y} ), $$

1d $$ \tan (2\Theta ) = \frac{{2\sigma_{xy} }}{{\sigma_{x} - \sigma_{y} }}, $$

and where, *f* defines the yield function, *R* is the distance between the stress point and the origin of coordinates, *c* is the soil cohesion, *φ*_max_ is the maximum peak internal friction angle. *n* and *β* are the anisotropic parameters, *n* = sin*φ*_min_/sin*φ*_max_ ranging from 0 to 1, with smaller value denoting more significant anisotropy of soil strength. When *n* = 1, Eq. (1) becomes the classical isotropic Mohr–Coulomb yield function. *β* is the direction angle corresponding to the maximum internal friction angle, namely, when *Θ* = *β*, sin*φ* = sin*φ*_max_. *β* ranges from 0 to π/4.

Based on the geometry of the ellipse, the variation of internal friction angles with the direction of principal stress can be defined as

2 $$ \sin \varphi (\Theta ) = \frac{{n \cdot \sin \varphi_{\max } }}{{\sqrt {n^{2} \cdot (\cos (2\Theta - 2\beta ))^{2} + (\sin (2\Theta - 2\beta ))^{2} } }} $$

**Figure 3:**
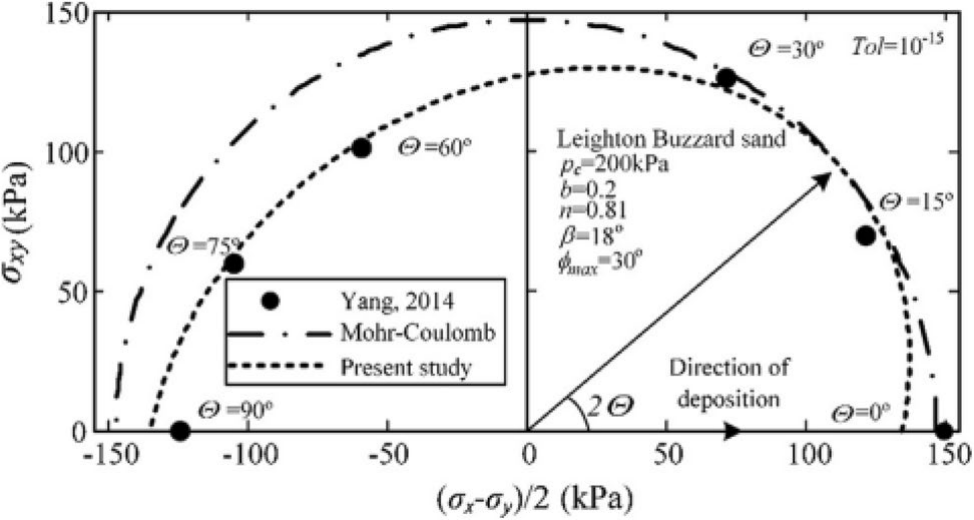
Validation of the newly proposed anisotropic yield criterion^14^.

For a specific soil with given *c*, *φ*_max_, *n*, *β*, the shear strength parameter sin*φ* variation with the major length of the ellipse direction *Θ,* which is shown in Fig. 3, has been previously studied by Yuan et al.^14^. This yield criterion has been validated by both experimental and micro-mechanical evidence, and its application to boundary value problems has been showcased^14^. For a specific slope stability problem, the soil strength parameters can be obtained by the direct shear test or the triaxial test, and the anisotropic parameters are got by the hollow cylinder torsional shear test. Based on these parameters, the upper bound LA or FEM can be used to analyze the slope stability.

## Upper bound theory and stability factor

### LA and stability factor

**Figure 4:**
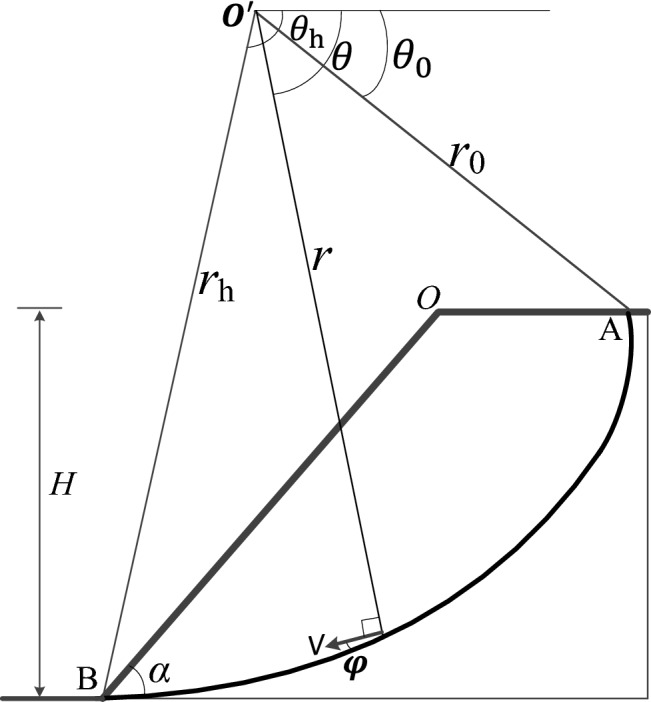
The failure mechanism for a slope.

The compatible velocity field for an upper-bound solution of slope stability is shown in Fig. 4. The region AOB rotates as a rigid body about the center of rotation O with the materials below the logarithmic surface AB remaining at rest. The equation for the logarithmic spiral slip surface is given by:

3 $$ r = r_{0} \exp \left[ {(\theta - \theta_{0} )\tan \varphi } \right] $$

where *r*_0_ is the length of chord OA, *θ*_0_ is the angle between the chord OA and the horizontal. Note that for an isotropic material, the internal friction angle is constant; however, in this manuscript, the anisotropic soil is discussed, the value of the internal friction angle is dependent on the values of *Θ*, *β*, and *n*, and it can be calculated by Eq. (2). The rate of external work for the region ABO is calculated by the algebraic summation:

4 $$ \dot{W} = \gamma r_{0}^{3} \omega \left( {f_{1} - f_{2} - f_{3} } \right) $$

where *γ* is the soil weight, *ω* is the rotational angular velocity of the block ABO, *f*_1_-*f*_3_ are the coefficients for the calculation of the soil weight work rate, which can be found in the literature^22^.

The internal dissipation of energy occurs along the slip surface AB. The total internal dissipation energy is obtained by the following integration:

5 $$ D = \int_{{\theta_{0} }}^{{\theta_{h} }} {c(V\cos \varphi )\frac{rd\theta }{{\cos \varphi }}} = \frac{{cr_{0}^{2} \omega }}{2\tan \varphi }\left\{ {\exp [2(\theta_{h} - \theta_{0} )\tan \varphi ] - 1} \right\} $$

Equating the external rate of work to the internal energy dissipation rate, yields:

6 $$ H = \frac{c}{\gamma }F(\theta_{h} ,\theta_{0} ) $$

where the function *F*(*θ*_h_, *θ*_0_) can be calculated as:

7 $$ F(\theta_{h} ,\theta_{0} ) = \frac{{\sin \beta \left\{ {\exp [2(\theta_{h} - \theta_{0} )\tan \varphi ] - 1} \right\}}}{{2\sin (\beta - \alpha )\tan \varphi (f_{1} - f_{2} - f_{3} )}}\left\{ {\sin \theta_{h} \exp [(\theta_{h} - \theta_{0} )\tan \varphi ] - \sin \theta_{0} } \right\} $$

Apparently, *F*(*θ*_h_, *θ*_0_) is a function of parameters *θ*_h_ and *θ*_0_, and its minimum value can be calculated using an optimization approach. Based on Eq. (6), the least upper bound of the critical height *H*_c_ is obtained when the minimum value of *F*(*θ*_h_, *θ*_0_) is obtained. Denoting *N*_s_ = min*F*(*θ*_h_, *θ*_0_), associating with Eq. (6), we have:

8 $$ N_{s} = \frac{{\gamma H_{c} }}{c} = \min F(\theta_{h} ,\theta_{0} ) $$

**Figure 5:**
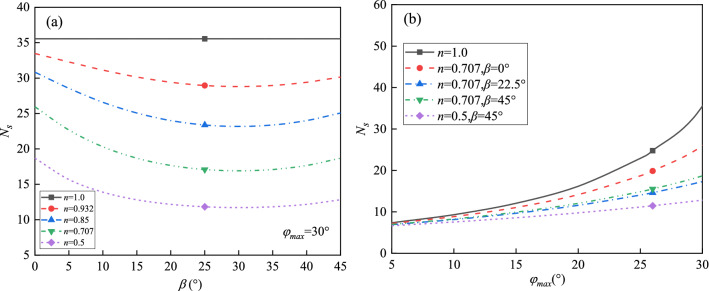
The effects of anisotropic parameters on stability factor (slope angle *α* = 45°): (**a**) stability factor *N*s versus *β*; (**b**) stability factor *N*s versus *φ*_max_.

*N*_s_ is a dimensionless number, also known as the stability factor of the slope. For a slope with specific isotropic strength parameters, the value of *N*_s_ depends on the slope angle *α* and the internal friction angle *φ*. In this case, the stability factor is also affected by the anisotropic coefficients *n* and *β*. The coefficient *Θ* is assumed to be 70° in the kinematic approach of limit analysis. The parametric study of the anisotropic coefficients on the stability factor of the slope is carried out in the following. Figure 5a shows that the decreasing value of parameter *n* results in the decrease in the stability factor. For a specific value of *n*, the stability factor decreases with the increasing parameter *β* until* β* approaches 25°, where the stability factor reaches the minimum value. Thereafter the stability factor increases gradually with the increasing parameter *β.* Note that when *n* = 1, the stability factor is constant even though the parameter *β* varies from 0° to 45°, because *n* = 1 denotes the isotropic condition. Not surprisingly, the stability factor increases with the increasing maximum internal friction angle *φ*_max_ regardless of whether the anisotropic condition is considered or not (Fig. 5b). Note that the differences between the isotropic and anisotropic results become pronounced with the increase in *φ*_max_.

**Figure 6:**
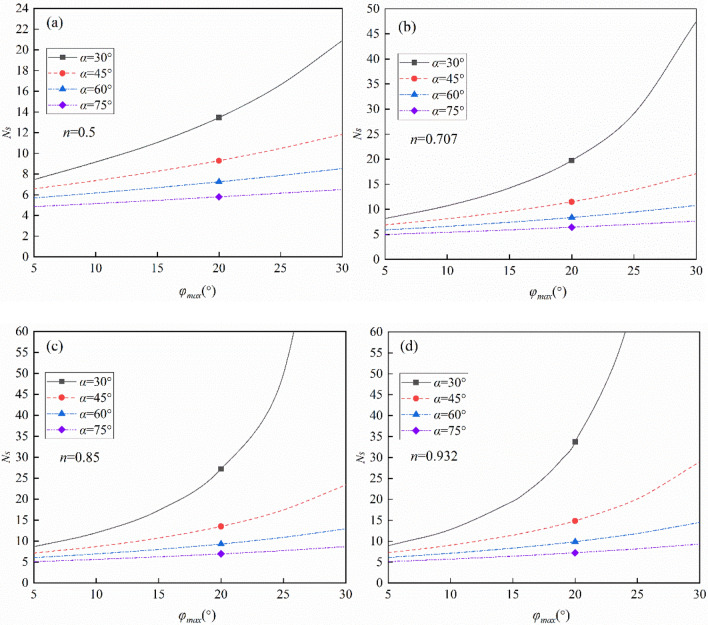
The effect of slope angle *α* on the stability factor (*β* = 25°): (**a**) *n* = 0.5, (**b**) *n* = 0.707, (**c**) *n* = 0.85, (**d**) *n* = 0.932.

When the anisotropic parameter *β* = 25°, the stability factor varies with the maximum internal friction angle *φ*_max_ for different slope angles, as shown in Fig. 6. The increasing parameter *n* has a significant effect on the stability factor for gentle slopes (*α* ≤ 45°), while a less obvious effect is observed in steep slopes (*α* ≥ 60°). For instance, when *φ*_max_ = 15°and *n* = 0.5, the stability factor for the slopes with the inclination angles of 30° and 75° are 11.04, 5.46, respectively. Whereas, the counterpart is 19.51 and 6.40 when *n* = 0.932. The growth rates are 77% and 17% for the gentle and steep slopes, respectively.

### The SSRA and safety factor

Based on the SSRA, the safety factor can be defined as:

9 $$ c^{\prime} = c/F_{s} $$

10 $$ \tan \varphi^{\prime} = \tan \varphi /F_{s} $$

where *c′* and *φ′* are the soil strength parameters necessary only to maintain the structure in limit equilibrium and sometimes referred to as the mobilized strength parameters.

Factored shear strength parameters *c′*, *φ′* are used to bring the slope to the point of failure. Substituting Eqs. (2), (9), and (10) into Eq. (6), the safety factor can be calculated. To avoid the iteration, the stability charts are presented herein for slopes with specific *γH*/*c*, *α*, *β*, and *n* in the following.

**Figure 7:**
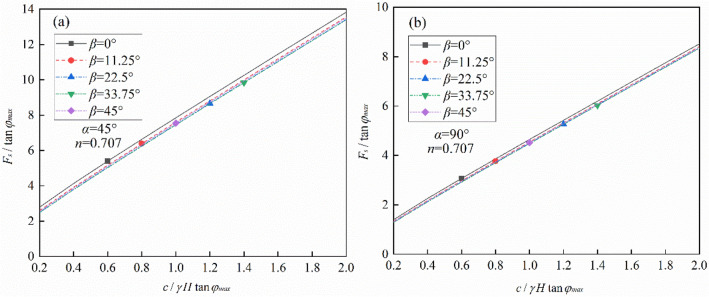
Stability chart for slopes considering strength anisotropy: (**a**) *α* = 45°, (**b**) *α* = 90°.

To make a quick estimate of the slope stability considering soil strength anisotropy, the stability chart is presented in Fig. 7. For a specific slope, the maximum value of *F*_s_ can be obtained with *β* = 0°. In addition, it is shown that for a specific slope with known values of *c*/(*γH*tan*φ*_max_) and slope angles *α*, the factor of safety of the slope can be obtained from Fig. 7 without any iteration. For example, for a slope inclined at 45°, with a height of 10 m, the soil unit weight is 18 kN/m^3^, the soil cohesion is 22 kPa, the maximum internal friction angle *φ*_max_ = 17°, and the anisotropic parameters are tested by lab testing (*n* = 0.707, *β* = 11.25°). It can be calculated that *c*/(*γH*tan*φ*_max_) = 0.4. We can get that *F*_s_/tan*φ*_max_ = 3.928 from the red line in Fig. 7a. Then, the factor of safety is calculated as *F*_s_ = 1.2.

## The numerical analysis procedure

It has been widely accepted that the slope stability is significantly affected by the strength anisotropy of soils^22,31^. To this end, a comparison between the influences of isotropic and anisotropic strength on the slip surface of slopes is sought. The SSRA associated with FEM was proposed by Zienkiewicz et al.^36^, and a good agreement with the slip circle solution was obtained for *c*-*φ* soil slopes*.* This approach was extended by many to slope stability analysis^37–40^, and is adopted here, with a summary of the procedure presented in what follows. The definition of the safety factor is the same as in Eqs. (9) and (10). An iteration process is required to establish the expression of *F*_s_. When the slope failure occurs, the factor *F*_*s*_ is the desired solution.

Slope stability analysis inherently refers to an elastoplastic problem. The quadrilateral isoparametric element is used for modelling in FEM. In the elastic regime, the Hooke’s law is considered, while in the plastic regime, the stress–strain relationship in its incremental form is given as:

12 $$ \dot{\user2{\sigma }} = {\Delta }{\varvec{\sigma}}_{e} - {\Delta }\lambda {\varvec{D}}\left( {\partial g/\partial {\varvec{\sigma}}} \right) $$

where Δ***σ***_e_ stands for the vector of elastic stresses, Δ***σ***_e_ = ***D***Δ**ε** (Δε is the strain rate); *D* is the stress–strain matrix; *g* is the plastic potential; and Δ**λ** can be calculated as

13 $$ \Delta {\varvec{\lambda}} = \frac{{{\varvec{D}}({{\partial f} \mathord{\left/ {\vphantom {{\partial f} {\partial {\varvec{\sigma}}}}} \right. \kern-\nulldelimiterspace} {\partial {\varvec{\sigma}}}})^{T} \Delta {\varvec{\varepsilon}}}}{{({{\partial f} \mathord{\left/ {\vphantom {{\partial f} {\partial {\varvec{\sigma}}}}} \right. \kern-\nulldelimiterspace} {\partial {\varvec{\sigma}}}})^{T} {\varvec{D}}({{\partial g} \mathord{\left/ {\vphantom {{\partial g} {\partial {\varvec{\sigma}}}}} \right. \kern-\nulldelimiterspace} {\partial {\varvec{\sigma}}}})}} $$

in which, *f* is the yield function. When the associated flow rule is applied, the expression of plastic potential *g* equals to that of the yield function *f*.

After the initial yielding is determined, the explicit modified Euler integration scheme with stress correction^34^ is applied to calculate the elastoplastic stress and strain. Strain hardening is not considered, and all the stresses and strains are integrated at the Gauss points. The Newton–Raphson method has been found to converge rapidly in FE codes. The displacement mutation of the characteristic point combining with the continuums of the plastic zone can be regarded as a reliable definition of slope failure. The procedure of the iteration algorithm used can be found in the literature^40^. The implementation of the proposed yield function into FE analysis faces computational challenges, mainly because of the gradient discontinuities at the tip or vertex of the yield surface. Note that, some recent researches shows that the artificial intelligence-based technology can offer more progressive analysis tool^41–43^, which no longer need to consider the gradient discontinuities problems. However, in this manuscript, the traditional processing procedure is utilized by removing singularities in the yield surface. Among these, the method proposed by Abbo and Sloan^44^ is adopted by introducing a parameter *a* = 0.05*c*cot*φ*_*max*_. This latter so called hyperbolic approach can be found for brevity in the Appendix.

## Case study

One numerical example of typical soil slope models is used to analyze the effects of strength anisotropy on slope stability. The geometry of the slopes and the material properties of the soils are adopted as in Dawson^45^. Parametric studies on the influence of the degree of the strength anisotropy, in terms of anisotropic parameters, on the slope stability problem are presented. Anisotropic parameters followed Yuan et al.^14^.

**Figure 8:**
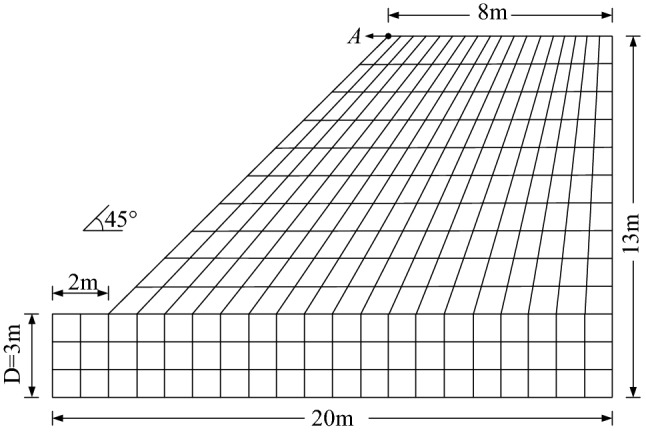
The discrete mesh of soil slope model.

A simple slope adopted from Dawson^45^ is shown in Fig. 8. The slope has a height of 10 m with a base of 3 m, and the inclination of the slope is 45°. The four-node quadrilateral isotropic element is utilized to discretize the slope model, with a total number of 330 elements and 369 nodes. On the left and right boundaries, vertical rollers are applied to restrict the horizontal displacement, while full fixity is applied at the base. The properties of the soil are listed in Table 1. It has been discussed by Griffith and Lane^37^ that the values of Young’s modulus and Poisson’s ratio have negligible influences on the predicted *F*_s_. Typical values are given to the soils, namely, *E* = 10,000 kN/m^2^, *υ* = 0.3. The selection for the anisotropic parameters *n* and *β* will be detailed in what follows. The associated normality flow rule for elastoplastic material is used. Note that the soil strength parameters in this manuscript should be the effective strength indexes. Further, in our constitutive model, the remolded soil is used. The measured shear strength parameters and anisotropic parameters show the characteristics of the remolded soil. For the natural structured soil, our constitutive model is not good at revealing its structural property.

**Table 1:** Material properties of the numerical model .

Unit weight *γ* (kN/m^3^)	Friction angle *φ*_*max*_ (°)	Cohesion *c* (kPa)	Young’s modulus *E* (kN/m^2^)	Poisson’s ratio *υ*
20	20	12.38	10,000	0.3

For the plane strain condition, the effective plastic strain (EPS) is calculated as:

14 $$ EPS = \sqrt {({2 \mathord{\left/ {\vphantom {2 3}} \right. \kern-\nulldelimiterspace} 3})(d{{\varvec{\upvarepsilon}}}_{p} )^{T} (d{{\varvec{\upvarepsilon}}}_{p} )} $$

where d***ε***_p_ is the plastic strain increment, and it is defined as:

15 $$ d{{\varvec{\upvarepsilon}}}_{p} = \lambda (\partial g/\partial {{\varvec{\upsigma}}}) $$

in which λ is the plastic multiplier, *g* is the plastic potential, **σ** is the stress tensor.

**Figure 9:**
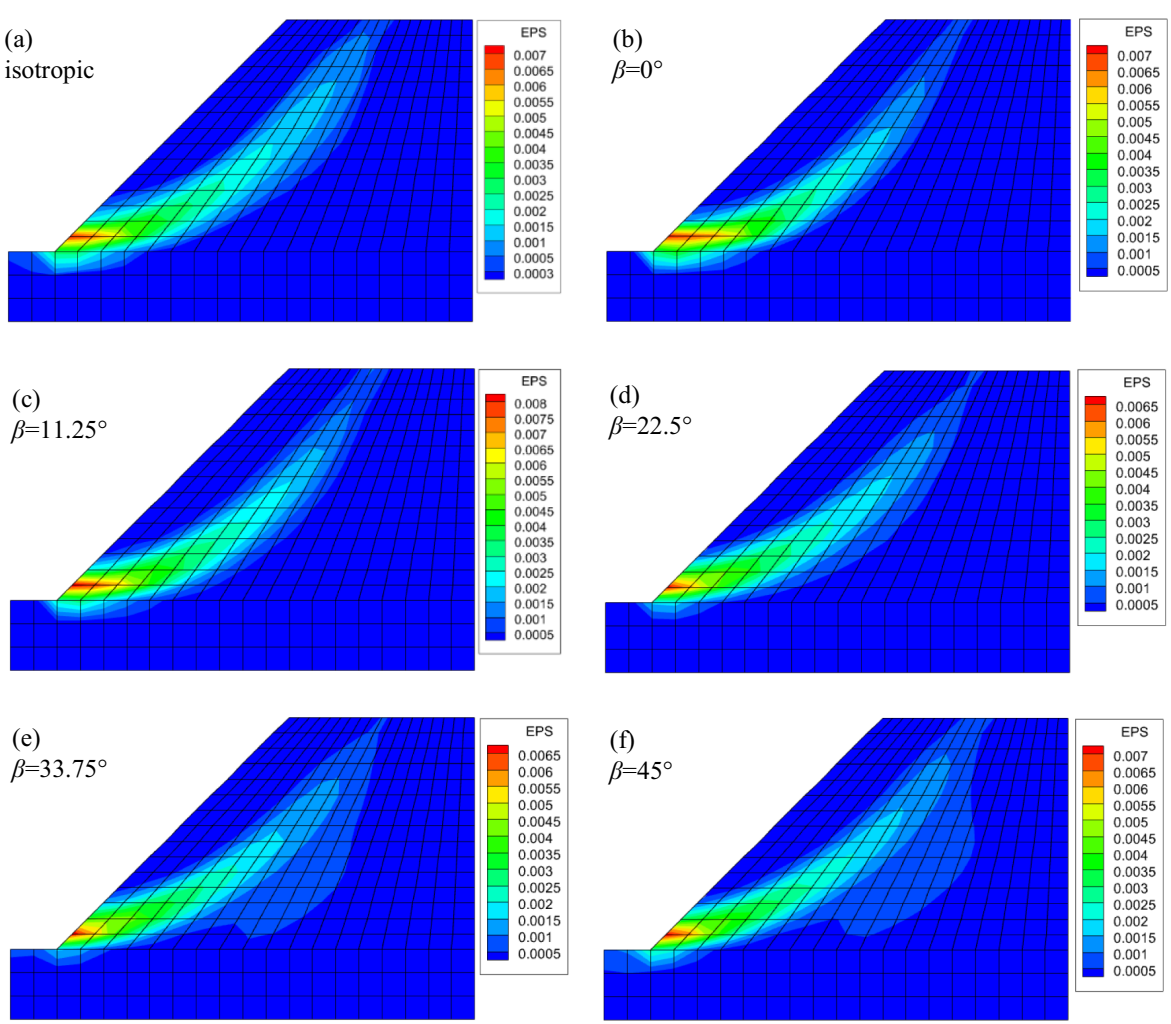
Effective plastic strain nephograms of slopes for isotropic and anisotropic [(**b**)–(**f**), *n* = 0.707] conditions: (**a**) isotropic(*n* = 1), *F*_s_ = 1.03; (**b**) *F*_s_ = 0.89; (**c**) *F*_s_ = 0.825; (**d**) *F*_s_ = 0.79; (**e**) *F*_s_ = 0.81; (**f**) *F*_s_ = 0.87.

The EPS nephograms of the slope for isotropic and anisotropic conditions are displayed in Fig. 9. Since the increasing shear strength reduction (SSR) method is used in the numerical simulation, the status of the slope varies from stable to unstable with the increasing SSR factor. When the plastic zone developed from the slope toe to the top, combining with the displacement mutation of the characteristic point (point A in Fig. 8), it can be regarded as the slope failure condition. In other words, the slope is under limit state condition. At this time, the calculated Max. EPS of the slope is relatively small. It has been previously calculated that the *F*_s_ of this slope is exactly 1.0 for homogeneous isotropic conditions through limit analysis^22^. Dawson et al^45^ worked out the *F*_s_ and estimated it to be between 1.02 and 1.03, by using FEM combined with SSRA. It has been demonstrated that the numerical results are slightly different than the limit analysis solutions for most of the times, but within reasonable bounds. In this paper, the calculated *F*_s_ of the isotropic slope is 1.03, which agrees well with the literature. Figures 4b–f show the anisotropic results. It is depicted that when *n* = 0.707, the *F*_s_ is always lower than 1.0. The trace of EPS, which can be regarded as the slip surface, varies with the value of anisotropic parameter *β*. With increasing *β*, the slip surface gradually moves away from the slope shoulder until approximately *β* = 22.5*°*, after which it starts returning closer to the shoulder. Figure 9 demonstrates that the consideration of strength anisotropy will lead to a more critical failure mechanism.

**Figure 10:**
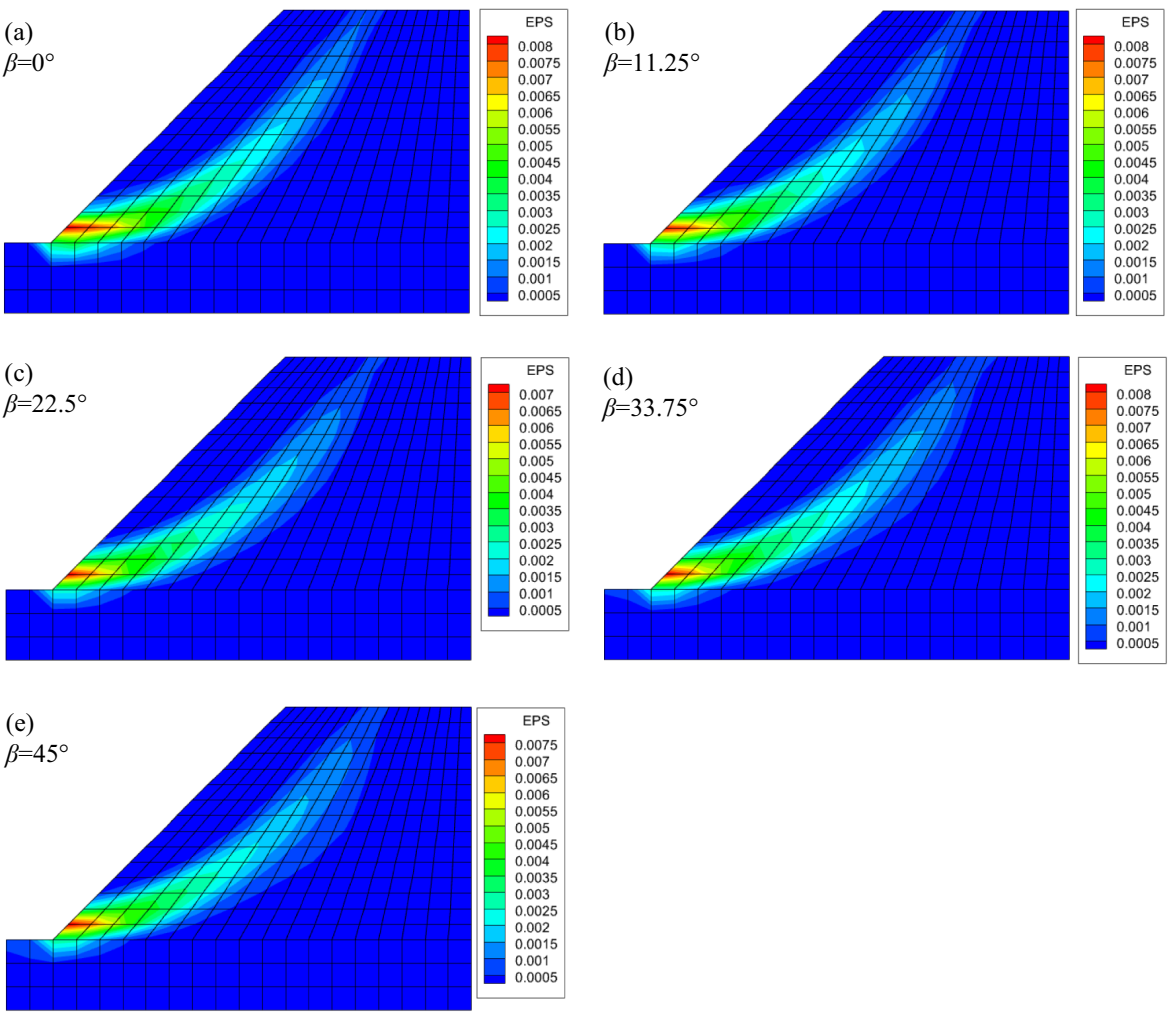
Effective plastic strain nephograms of slopes for anisotropic conditions (*n* = 0.85): (**a**) *F*_s_ = 0.97; (**b**) *F*_s_ = 0.94; (**c**) *F*_s_ = 0.92; (**d**) *F*_s_ = 0.94; (**e**) *F*_s_ = 0.96.

**Figure 11:**
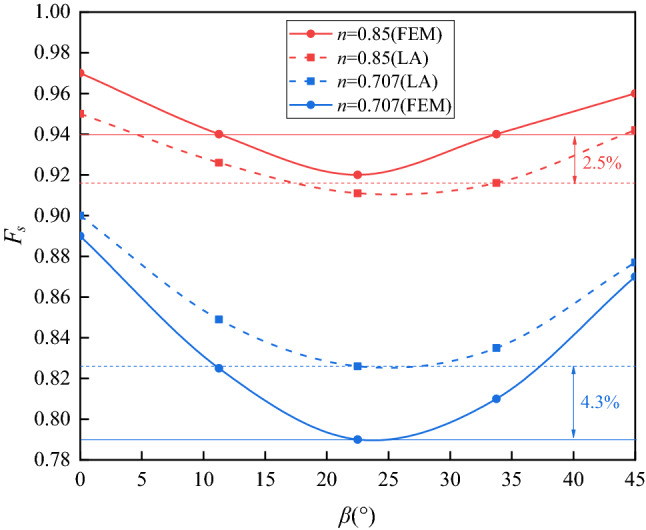
Factor of safety of slopes against the anisotropic parameter *β*.

When the parameter *n* = 0.85, the EPS nephograms are displayed in Fig. 10. The strength anisotropy results again in a lower *F*_s_ than in those from the isotropic solution. The *F*_s_ values in Fig. 10 are all very close to the isotropic solution, i.e., 1.03, with the *β* values varying equidistantly from 0° to 45°. In addition, comparing with Fig. 9, there is not much difference relevant to the shape of the slip surface for different *β* values. This is because the *n* value in Fig. 10 is larger than that in Fig. 9. As mentioned before, the closer the *n* value approaches to 1, the greater the anisotropic effect diminishes. The *F*_s_ of slopes for different *β* values are collected in Fig. 11. It is shown that for a specific value of *n*, the *F*_s_ of slopes decreases with the increase in *β* until *β* = 22.5°, for the actual *β* sampling considered. There the *F*_s_ reaches a nominal minimum value, and then increases gradually for even higher *β*. The variation of *F*_s_ follows the development of the internal friction angle *φ*. The initial strength anisotropy is developed by the varying of internal friction angles with the principal stress direction, like a rotational ellipse, following Booker and Davis^35^, Yuan et al.^25^. Here, *n* and *β* are two parameters that are introduced to describe the initial strength anisotropy of soils. Actually, the parameter *β*, together with the parameter *n*, governs the size of the anisotropic yield curve. The parameter *β* refers to an angle when the major principal stress (corresponding to the case of the maximum peak internal friction angle) is inclined to the deposition direction. Since the evolution of *β* is not linear with loading, there exists a critical value, before which the internal friction angle decreases with an increase in the value of *β*. However, the internal friction angle increases with increasing *β* after the critical value. Hence, the value of the safety factor of the stability slope stability coefficient first decreases and then increases as shown in Fig. 11*.* Figure 11 also displays that for a given *β* value, a smaller *n* value results in a lower *F*_s_. This is because the *n* value defines the shape of the anisotropic yield surface, which is an ellipse. The larger the *n* value is, the greater the shape of the yield surface approaches a circle, namely, and the soil performs closer to the less onerous isotropic condition. Generally, if the strength anisotropy is neglected, the overestimation of *F*_s_ can get up to 32.9% for *n* = 0.707; and 14.1% for when *n* = 0.85. In addition, it is displayed that the results obtained by LA and FEM show the same trends. The two methods lead to different *F*_s_ values, but the discrepancy is quite small. When *n* = 0.707, the maximum difference of *F*_s_ is only 4.3% in the presented case. Further, this difference decreases with the increasing *n* value. For instance, when *n* = 0.85, the maximum difference of *F*_s_ between the two methods decreases to 2.5%. Two reasons can be drawn for the explanation of this discrepancy: firstly, in the upper bound LA method, since the stresses in the soil is not considered, the value of *Θ* is defined as a constant, i.e., 70°. Secondly, FEM and LA are based on different algorithms, this leads to different results.

## Conclusions

Soils, although having their anisotropic mechanical performance recognized, they are mostly regarded as isotropic in traditional slope stability analyses. This deficiency may result in the overestimation of factors of safety. For this reason, slope stability analyses considering the strength anisotropy have been recently carried out to capture explicit anisotropy effects. However, most of them focus on undrained soils. In this paper, an anisotropic yield criterion was introduced to analyze the influence of strength anisotropy on generic *c*–*φ* soil type slopes.

The anisotropic yield criterion was combined with the kinematic method of limit analysis and FEM respectively to analyze the slope stability. The stability charts were presented to make a quick estimation of the *F*_s_. The values of *F*_s_ obtained by LA and FEM showed high agreement one another. The slope stability was largely overestimated if the strength anisotropy was ignored. For instance, the maximum overestimation can reach up to 32.9% for the presented case. The parametric analysis shows that the increasing parameter *n* resulted in a pronounced increment in the stability factor for steep slopes (α ≥ 60°), whereas, this increment was less sensitive for gentle slopes (α ≤ 45°). In addition, the presented case showed that for a given *n* value, a minimum peak factor of safety was obtained when *β* = 22.5°.

## Supplementary Information

Supplementary Information.

## Data Availability

Some or all data, models that support the findings of this study are available from the corresponding author upon reasonable request.
