# Supplementary material for: Slope stability analysis considering the strength anisotropy of c-φ soil
Source: Sci Rep. 2022 Nov 1;12:18372. doi: 10.1038/s41598-022-20819-y (PMC9626556; doi:10.1038/s41598-022-20819-y)
Supplement: Supplementary file 1 — Supplementary Information. [file 41598_2022_20819_MOESM1_ESM.docx]

**Appendix**

In displacement-based FE analysis, the traditional Mohr-Coulomb yield function presents a number of computational difficulties due to the gradient discontinuities which occur at the tip of the yield surface and the edges of the hexagonal yield surface pyramid. To avoid these computational difficulties, a number of methods have been proposed to remove the associated gradient singularities. In the present paper, the method proposed by Abbo and Sloan (1995) has been adopted, and this is briefly described below.

A hyperbolic approximation in the meridional plane is used to eliminate the tip singularity. In ($\sigma_{m}$,$\bar{\sigma}$) space, the hyperbolic approximation to the Mohr-Coulomb yield criterion is shown in Fig. A1

Fig. A1 Hyperbolic approximation to Mohr-Coulomb yield criterion^[36]^.

The equation of the hyperbola in Fig.A1 can be mathematically described by:

 (A1)

in which,

$\sigma_{m}=\frac{1}{3}(\sigma_{x}+\sigma_{y}+\sigma_{z})$ (A2)

$J_{2}=\frac{1}{2}(s_{x}^{2}+s_{y}^{2}+s_{z}^{2})+\tau_{xy}^{2}+\tau_{yz}^{2}+\tau_{xz}^{2}$ (A3)

 (A4)

 (A5)

$\begin{matrix} sign(\theta)=+1 (\theta\geq0^{^{\circ}}) \\ sign(\theta)=-1 (\theta<0^{^{\circ}}) \end{matrix}$ (A6)

We have used Eq.(A1) to model the Mohr-Coulomb yield function. Apparently in Fig.A1, *a* is the distance of the Mohr-Coulomb yield surface tip and the hyperbola tip, and a smaller value of *a* represents a closer approximation of the Mohr-Coulomb yield surface. The hyperbolic yield surface with various *a* values is displayed in Fig. A2.

Fig. A2 Hyperbolic approximation to Mohr-Coulomb meridional section^[34]^.

More details of the hyperbolic yield surface can be found in Abbo^[34]^ and He et al^[40]^.
